# Study on the Temperature-Dependence of the Modulus of LSAM-50 Pavement Materials

**DOI:** 10.3390/ma18112606

**Published:** 2025-06-03

**Authors:** Yingjun Jiang, Jiangang Xu, Hongjiang Zhang, Yingchao Liang, Jinpeng Du, Di Wu

**Affiliations:** 1 Key Laboratory for Special Area Highway Engineering of Ministry of Education, Chang’an University, Xi’an 710064, China; 2022121202@chd.edu.cn; 2 Shaanxi Jiao Kong Municipal Road & Bridge Group Co., Ltd., Xi’an 710075, China; 15829932171@163.com (H.Z.); dujinpeng074@163.com (J.D.); wud0754@163.com (D.W.)

**Keywords:** LSAM-50 pavement materials, temperature, resilient modulus, dynamics modulus, master curve, dynamic modulus prediction model

## Abstract

To investigate the temperature dependence of the modulus of LSAM-50 flexible base asphalt pavement (LSAM-50 pavement) materials, specifically SMA-13, AC-20, and LSAM-50. The effects of temperature on the modulus of LSAM-50 pavement materials were investigated, and a temperature-dependent model of resilient modulus was established. A dynamic modulus master curve was constructed based on a generalized logarithmic Sigmoidal model. The correlation between the resilient modulus and dynamic modulus was studied, and a multiple linear regression model was developed to describe the relationship between the dynamic modulus and resilient modulus, temperature, and loading frequency. The results show that the resilient modulus and dynamic modulus gradually decrease with the increase in temperature and then tend to stabilize. The resilient modulus of LSAM-50 is higher than that of SMA-13 and AC-20 in the entire temperature range, and the dynamic modulus of LSAM-50 is higher than that of SMA-13 and AC-20 in the high-temperature range. The correlation coefficients (*R*^2^) of the established resilient modulus and dynamic modulus estimation models are greater than 0.97 and 0.94, respectively.

## 1. Introduction

Long-life asphalt pavement has become an important research topic in the field of road engineering. Numerous practical engineering studies have demonstrated that a thick asphalt layer or a full-thickness asphalt pavement structure is less prone to structural damage from the bottom to the top. Consequently, the service life of such pavements is generally longer [1,2,3,4]. The LSAM-50 pavement developed in the early stage of the research group is a full-thickness asphalt pavement, and its typical structure is 30~40 mm flexible base layer +6 mm joint layer +4 mm surface layer. LSAM-50 has the advantages of strength, rutting resistance, economy, etc., and can be used as the flexible base layer of LSAM-50 pavement [5,6,7,8], while SMA-13 and AC-20 can be used as the surface layer and joint layer of LSAM-50 pavement, respectively.

Asphalt mixtures are typical temperature-sensitive materials, and their performance characterization has significant temperature dependence. Qian et al. [9] conducted direct tensile, uniaxial compression, and shear resistance tests on asphalt mixtures at various temperatures, and proposed a strength model that accounts for the effect of temperature based on the unified strength theory. The results showed that the strength prediction model, which incorporates temperature effects, demonstrated a high correlation with the actual. Pérez-Jiménez et al. [10] investigated the effects of temperature and load coupling on the low-temperature cracking resistance of asphalt mixtures. The results showed that asphalt mixtures lose viscosity at −15 °C and exhibit significant rigidity. Boussabnia et al. [11] found that the stiffness of mixtures increases at low temperatures and the fatigue life is shorter. Omran et al. [12] found that when the temperature increases from 40 °C to 50 °C, the permanent deformation of asphalt mixtures will increase by 61% and creep modulus decreases by 34%. Jiang et al. [13] carried out repeated loading tests at different temperatures with a variety of perimeter pressures and found that the higher the temperature, the greater the permanent deformation of the asphalt mixture and the stronger the load sensitivity.

The research conducted by the aforementioned scholars on the impact of temperature on the performance of asphalt mixtures has significantly advanced the quality of road construction and the prevention of pavement damage. A critical aspect of this research is how to integrate the temperature dependence of material performance with practical engineering considerations. The modulus value plays a pivotal role, as it serves as the foundation for pavement load response and structural design. Enhancing the reasonableness and accuracy of the modulus value is essential for optimizing the characteristics of road construction materials. This improvement can help mitigate issues, such as pavement rutting and cracking, which may occur prematurely within the design life of the pavement [14,15,16].

The resilient modulus characterizes the ability of the specimen to resist vertical deformation and is defined as the relationship between the load strength and the resilient deformation of the specimen under the action of loading and unloading in the vertical direction step by step. However, the resilient modulus test typically employs fixed temperature and load conditions, which do not accurately represent the temperature and stress conditions of asphalt mixtures in actual pavements. As the research advances and pavement design methods evolve, the dynamic modulus—which more accurately reflects the true mechanical properties of pavement materials—has garnered increasing attention from researchers. The American Association of State Highway and Transportation Officials (AASHTO) pavement design guidelines incorporate the dynamic modulus as a key parameter in pavement design [17]. AASHTO utilizes asphalt pavement core specimens to conduct repeated-loading indirect tensile tests, establishing the relationship between dynamic modulus and temperature. This relationship allows for the interpolation of the modulus value of the mixture under varying temperature conditions [18,19]. Christensen et al. [20] established a dynamic modulus prediction model that can be utilized to predict the dynamic modulus of the mixture based on the mineral gap rate and asphalt saturation. Ali et al. [21] investigated the effects of temperature, loading frequency, and mineral gap rate on the dynamic modulus of asphalt mixtures, establishing a nonlinear regression model that relates the dynamic modulus to these influencing factors.

To characterize the dynamic viscoelastic mechanical properties of asphalt mixtures and to obtain their dynamic properties across a broader range of temperatures and frequencies, some researchers have utilized the existing test data on dynamic modulus to construct a dynamic modulus master curve based on the principle of time–temperature equivalence. The sigmoidal model for fitting dynamic modulus master curves is proposed by the NCHRP Project A-37 [22,23]:

$$\mathrm{lg}\left|E^{*}\right|=\delta +\frac{\left(\alpha -\delta \right)}{1+e^{\beta +\gamma \mathrm{lg}fr}}.$$

Zhao et al. [24] proposed a horizontal shift factor model based on the sigmoidal model to simulate the effects of circumferential pressure and loading frequency:

$$\mathrm{lg}f_{r}=\mathrm{lg}f+\mathrm{lg}\alpha_{T}.$$

Sakhaeifar et al. [25] conducted function fitting using the least squares method. The dynamic modulus master curve is plotted, effectively capturing the dynamic viscoelastic properties of asphalt mixtures. Pablo et al. [26] tested the dynamic modulus of asphalt mixtures at five various temperatures and loading frequencies, constructing dynamic modulus master curves based on various fitting models. Some scholars have also established dynamic modulus master curves for asphalt mixtures under varying humidity levels, degrees of aging, and wet/dry cycle conditions. These curves are used to evaluate the effects of humidity, aging, and the number of wet/dry cycles on the dynamic modulus of asphalt mixtures [27,28,29]. In addition, constructing a dynamic modulus master curve is particularly relevant for studying the dynamic modulus of cold, recycled, asphalt mixtures [30].

Most of the studies on modulus mentioned above have been conducted on asphalt mixtures with a nominal maximum particle size of D ≤ 37.5 mm. However, the study of the modulus temperature dependence of LSAM-50 has not been reported. Furthermore, compared to semi-rigid base asphalt pavements, LSAM-50 pavements feature a thicker asphalt mixture layer, leading to pavement load responses and structural design that exhibit stronger temperature dependence. In light of this, this paper examines three asphalt mixtures commonly used in the typical structure of LSAM-50 pavements: LSAM-50, AC-20, and SMA-13. This study investigates the effects of temperature and nominal maximum particle size on the resilient modulus and establishes a temperature-dependent model for the resilient modulus. Additionally, it explores the effects of temperature, loading frequency, and nominal maximum particle size on the dynamic modulus and constructs a master curve for the dynamic modulus. Furthermore, the correlation between the resilient modulus and dynamic modulus is analyzed, leading to the establishment of a multiple linear regression model based on dynamic modulus, resilient modulus, temperature, and loading frequency. The concept of the modulus from static is of significant importance in guiding the design of roads in solid engineering.

## 2. Materials and Methods

### 2.1. Materials

Zhejiang Ningbo Donghai brand 70# base asphalt is utilized in LSAM-50 and AC-20, while the same brand’s styrene–butadiene–styrene (SBS) modified asphalt is employed in SMA-13. The technical properties of both the 70# base asphalt and the SBS-modified asphalt are shown in Table 1.

### 2.2. Mineral Grades

The mineral gradation of SMA-13, AC-20, and LSAM-50 is strong, embedded, skeleton, compact gradation, which is derived from the group’s previous research, as shown in Table 2.

### 2.3. Test Program

For the resilient modulus test, the test temperatures chosen were −15 °C, −10 °C, −5 °C, 0 °C, 5 °C, 15 °C, 20 °C, 30 °C, 45 °C, 60 °C, and 75 °C. For the dynamic modulus test, the test temperatures chosen were −15 °C, −10 °C, 0 °C, 10 °C, 20 °C, 30 °C, 40 °C, 50 °C, and 60 °C, and the loading frequencies chosen were 25 Hz, 20 Hz, 10 Hz, 5 Hz, 1 Hz, 0.5 Hz, and 0.1 Hz.

### 2.4. Specimen Sizes and Test Methods

#### 2.4.1. Specimen Sizes

For the resilient modulus test, the SMA-13 and AC-20 specimens had dimensions of φ 100 ± 2 mm × h 100 ± 2 mm, while the LSAM-50 specimens measured φ 200 ± 2 mm × h 160 ± 2 mm. For the dynamic modulus test, the SMA-13, AC-20, and LSAM-50 specimens were φ 102 ± 2 mm × h 150 ± 2.5 mm. The SMA-13 and AC-20 specimens were cored and cut from φ 150 mm × h 170 mm compacted samples, while the LSAM-50 specimens were φ 200 mm × h 160 mm, also obtained from coring and cutting the compacted sample of φ 200 mm × h 160 mm.

#### 2.4.2. Test Methods

**Figure 1:**
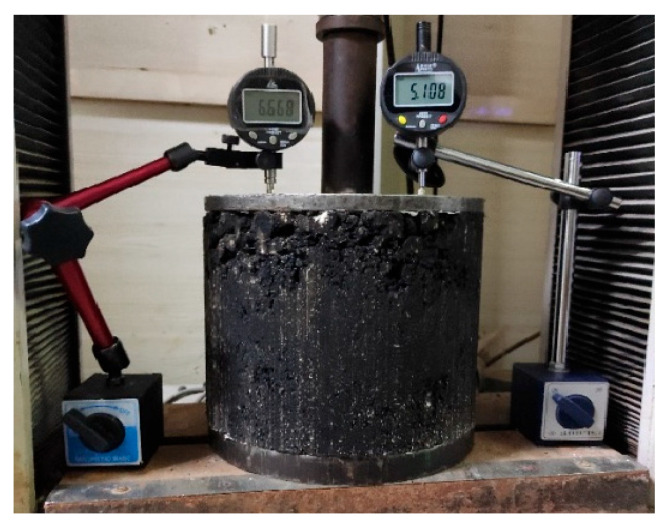
Resilient modulus test.

**Figure 2:**
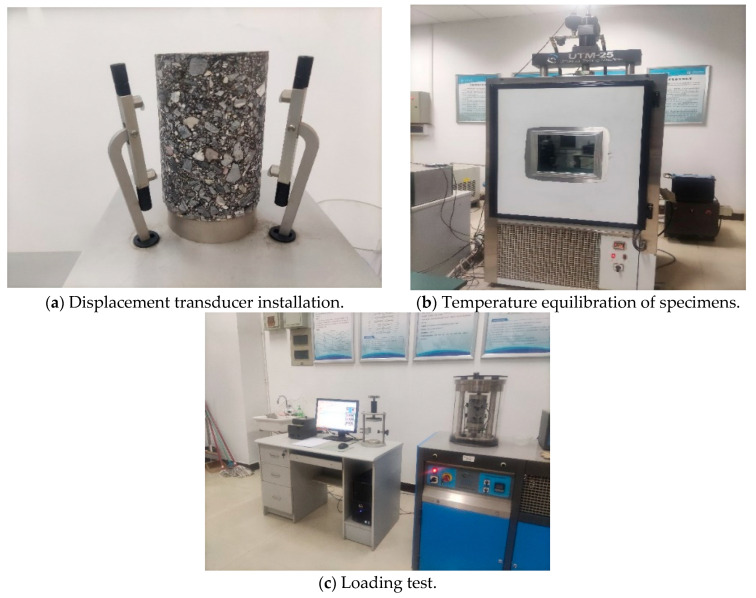
Dynamic modulus test.

(1) The resilient modulus test was conducted in accordance with T0713-2000 of the Test Methods for Asphalt and Asphalt Mixtures in Highway Engineering (JTG E20-2011), utilizing a loading rate of 2 mm/min. The testing procedure is shown in Figure 1. The formula is shown in Equation (1).
  (1) $$E_{c}=\frac{q_{5}\times h}{\Delta L_{5}}.$$
  where *E_c_* represents the resilient modulus; $q_{5}$ represents the compressive force under a level 5 load (MPa); *h* represents the axial height of the specimen (mm); and $\Delta L_{5}$. epresents the resilient deformation corrected to the origin at a level 5 load (mm).
(2) The dynamic modulus test was conducted in accordance with T0738-2000 of the Test Methods for Asphalt and Asphalt Mixtures in Highway Engineering (JTG E20-2011). The testing procedure is shown in Figure 2. The specimens were placed in an environmental chamber set to a specified test temperature with an accuracy of ±0.5 °C and maintained at a constant temperature for 4 h to 5 h until the desired test temperature was achieved, as shown in Figure 2b. During the testing process, the specimens were subjected to axial sine-wave loads at various test temperatures, ranging from a high to low frequency. The determination of each load frequency corresponded to the number of repetitions of the load under axial compressive stress and axial compressive strain, as shown in Figure 2c. The formula is shown in Equation (2):
  (2) $$\left|E*\right|=\frac{\sigma_{0}}{\varepsilon_{0}}.$$
  where E* represents the dynamic modulus (MPa), $\sigma_{0}$ represents the axial stress amplitude (MPa), and $\varepsilon_{0}$ represents the axial strain amplitude (mm/mm).

The resilient modulus and dynamic modulus tests were conducted at each temperature using four valid specimens. If the measured value deviated from the average value by more than 1.46 times the standard deviation, the measured value was discarded. The average results from four parallel specimens were calculated for each test condition.

## 3. Study on the Temperature Dependence of the Resilient Modulus in LSAM-50 Pavement Materials

### 3.1. Resilient Modulus Test Results

**Figure 3:**
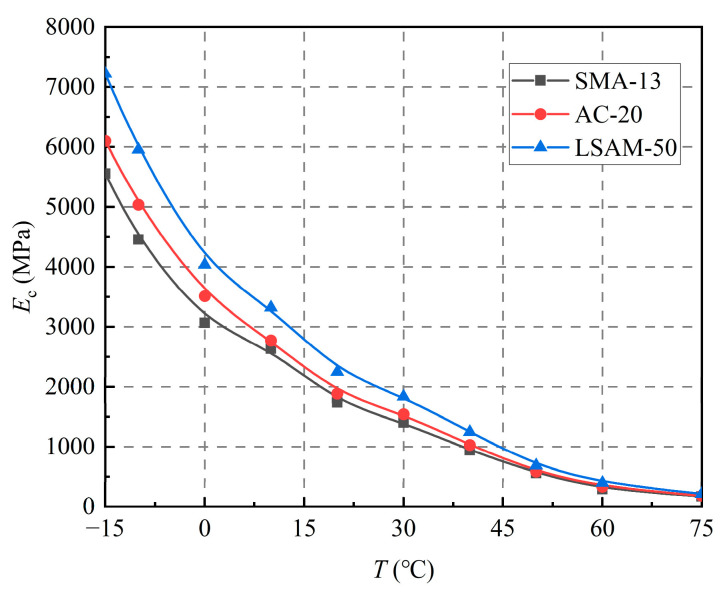
Effect of temperature and nominal maximum particle size on the resilient modulus.

Figure 3 illustrates the variation in the resilient modulus (*E*_c_) of SMA-13, AC-20, and LSAM-50 with temperature and nominal maximum particle size.

### 3.2. Analysis of Factors Affecting the Resilient Modulus

#### 3.2.1. Effect of Temperature

As shown in Figure 3, all three LSAM-50 pavement materials (SMA-13, AC-20, and LSAM-50) exhibit similar decreasing trends in the resilient modulus as the temperature rises; however, the rate of decline gradually slows, with 20 °C serving as the demarcation point.

Below 20 °C, a significant decrease in the resilient modulus is observed. As the temperature increases from −15 °C to 20 °C, the modulus variation in SMA-13, AC-20, and LSAM-50 accounts for approximately 75% of their total modulus changes throughout the entire temperature range.

Above 20 °C, the modulus decreases at a significantly slower rate. For the temperature increase from 40 °C to 75 °C, the changes in modulus for these mixtures account for only about 12% of the total variation across the entire temperature spectrum. The strength of asphalt mixtures can be attributed to the cohesive forces between aggregates, internal frictional resistance, and the binding properties of the asphalt.

When the temperature is low, the asphalt mixture enters an elastic state, where its strength is primarily derived from the adhesive properties of the asphalt. The strength of the asphalt increases, while its mobility decreases. Consequently, the strain induced in the mixture under load can be almost completely recovered.

As the temperature rises, the state of the asphalt and its physical and mechanical properties undergo significant changes. These changes are characterized by an increase in penetration and mobility, accompanied by a decrease in strength and viscosity. The asphalt mixture transitions from an elastic state to viscoelasticity and eventually to viscoplasticity. At elevated temperatures, when external forces are applied, asphalt mixtures experience substantial plastic deformation, leading to a lower resilient modulus for high-temperature asphalt mixtures [31].

When the temperature exceeds 40 °C, it approaches the softening point of asphalt, significantly diminishing the bonding effect of the material. Consequently, the strength of the mixture primarily relies on the interlocking forces between the aggregates and the internal friction resistance. At elevated temperatures, the resilient modulus of the asphalt mixture is notably reduced. The skeletal composition of the inter-mineral structure determines the mixture’s capacity to withstand external loads and deformation.

#### 3.2.2. Effect of Nominal Maximum Particle Size

As shown in Figure 3, the nominal maximum particle size significantly influences the resilient modulus. At any given temperature, the resilient modulus of the three LSAM-50 pavement materials ranks from largest to smallest as follows: LSAM-50, AC-20, and SMA-13. As the temperature increases, the resilient modulus of these three LSAM-50 pavement materials tends to converge. At a temperature of 60 °C, the resilient modulus of LSAM-50 is 1.20 times that of AC-20 and 1.37 times that of SMA-13, indicating that the advantage of LSAM-50 in terms of resilient modulus is particularly pronounced.

The analysis indicates that as the temperature rises, asphalt approaches its softening point, which weakens its bonding properties. The strength of the mixture primarily relies on the interlocking of coarse aggregates within the skeleton structure formed by embedded extrusion. This larger size allows the aggregates to interlock more effectively, resulting in a higher intensity of skeleton strength. Consequently, this leads to an increased resilient modulus.

### 3.3. Study on the Temperature-Dependent Resilient Modulus Model

(1) Model function

**Figure 4:**
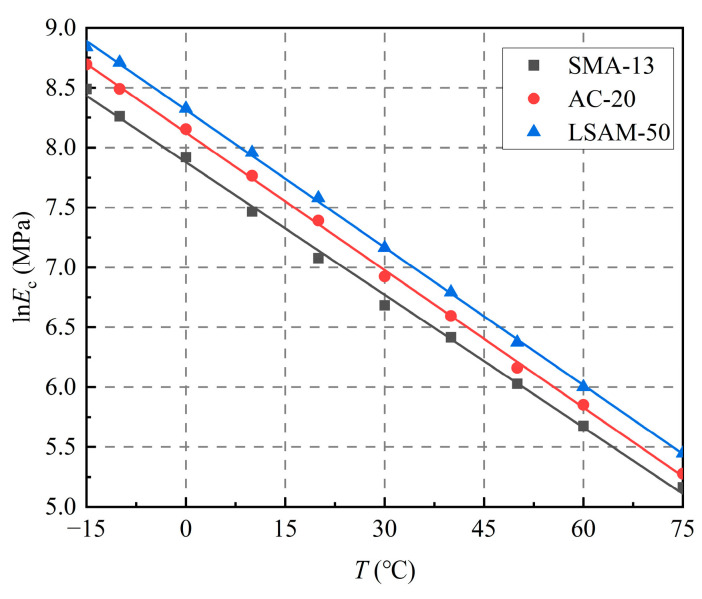
Relationship curve of ln*E*c versus temperature (*T*).

As shown in Figure 3, the resilient modulus consistently decreases with increasing temperature. Initially, the rate of decrease is rapid, followed by a gradual decline that resembles the growth pattern of a logarithmic function. The ln*E*_c_~*T* relationship curve is shown in Figure 4.

As shown in Figure 4, the natural logarithm of *E*_c_ (ln*E*_c_) demonstrates a linear correlation with temperature (*T*), as shown in Equation (3):

(3) $$\ln E_{cT}=a+bT.$$

The data show an improved fit with the logarithmic regression than with the alternatives. Taking the exponential of both sides of Equation (3), the temperature-dependent resilient modulus model is derived, as shown in Equation (4).

(4) $$E_{cT}=e^{a+bT}.$$

where *E*_cT_ represents the resilient modulus at a temperature of *T* °C (MPa), and *a* and *b* are the regression coefficients.

(2) Regression coefficient (a)

Let (*T* = 0). Substituting this value into Equation (4) results in Equation (5):

(5) $$E_{c0}=e^{a}.$$

Then, Equation (4) is transformed into Equation (6):

(6) $$E_{cT}=E_{c0}e^{bT}.$$

The three LSAM-50 pavement materials, *E*_c0_, are shown in Table 3.

(3) Regression coefficient (b)

Equation (6) is used to fit the data presented in Table 4, and the regression coefficient (*b*) along with the correlation coefficient (*R*^2^) are displayed in Table 4.

As shown in Table 4, Equation (6) more accurately characterizes the resilient modulus in relation to temperature changes, with *R*^2^ values exceeding 0.97. By substituting the regression coefficient (*b*) into Equation (4), we can derive the temperature-dependent models for the resilient modulus of SMA-13, AC-20, and LSAM-50, as shown in Equation (7):

(7) $$E_{cT}=E_{c0}e^{-0.039T}.$$

(4) Model reliability verification

**Figure 5:**
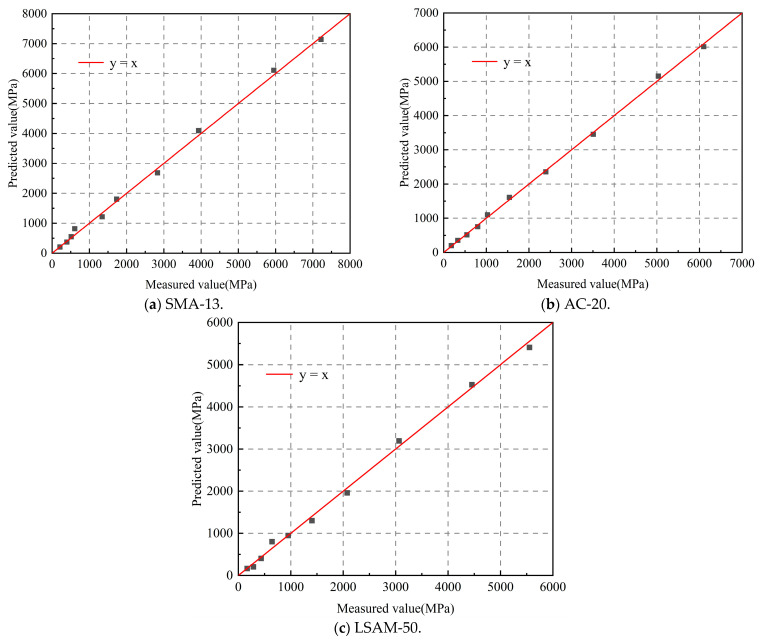
Scatter plot comparing the predicted and measured resilient modulus for various LSAM-50 pavement materials.

According to Equation (7), the predicted values of the resilient modulus have been obtained. A scatter plot is created to compare the predicted and measured values, with the measured values represented on the horizontal axis and the predicted values on the vertical axis, as shown in Figure 5.

As shown in Figure 5, the scatter plot depicting the predicted and measured values of the resilient modulus for the three LSAM-50 pavement materials aligns closely with the line y = x. This alignment signifies a strong correlation between the predicted and measured resilient modulus values, indicating that the predicted values can be effectively utilized to estimate the resilient modulus, as outlined in Equation (7).

Equation (7) demonstrates that the asphalt mixture’s resilient modulus at any temperature can be predicted through 0 °C testing, although achieving accurate measurements under such low-temperature conditions requires precise environmental controls.

The current research typically focuses on the resilient modulus at a conventional temperature of 20 °C. Therefore, it is essential to establish the relationship between *E*_c0_ and *E*_c20_. According to Equation (7), we can derive that *E*_c0_ = 2.2*E*_c20_. This relationship enables us to develop a temperature-dependent model for the resilient modulus of the asphalt mixture based on the resilient modulus at 20 °C, as shown in Equation (8).

(8) $$E_{cT}=2.2e^{-0.039T}E_{c20}.$$

where *E*_c20_ represents the resilient modulus at 20 °C (MPa), as determined through testing.

The formula is only applicable to the three LSAM-50 pavement materials in this paper; whether it can be applied to other types of asphalt mixtures need to be further verified by experiments.

## 4. Study on the Temperature Dependence of the Dynamic Modulus in LSAM-50 Pavement Materials

### 4.1. Dynamic Modulus Test Results

Table 5 presents the measured values of the dynamic modulus (E*) for SMA-13, AC-20, and LSAM-50.

### 4.2. Analysis of Factors Affecting the Dynamic Modulus

#### 4.2.1. Effect of Temperature

**Figure 6:**
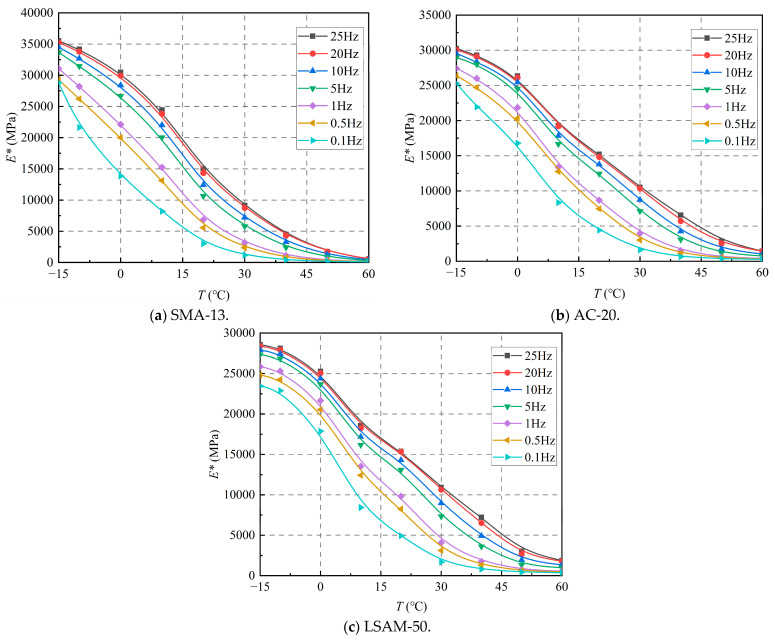
Effect of temperature on dynamic modulus for various LSAM-50 pavement materials.

The effect of temperature on the dynamic modulus is shown in Figure 6.

As shown in Figure 6, the dynamic modulus of the three LSAM-50 pavement materials exhibits a similar decreasing trend with increasing temperature across all loading frequencies. When the temperature is below 20 °C, the dynamic modulus decreases sharply. Conversely, when the temperature exceeds 40 °C, the rate of decrease in the dynamic modulus slows significantly. This phenomenon occurs because, as the temperature rises, the viscoplasticity of the asphalt increases, leading to a substantial unrecoverable plastic deformation under load. By the time the temperature reaches 40 °C, it approaches the softening point of the asphalt, resulting in a marked increase in viscoplasticity. Consequently, the deformation experienced under load transitions to predominantly unrecoverable plastic permanent deformation, which accounts for the significant reduction in the rate of dynamic modulus decrease.

#### 4.2.2. Effect of Loading Frequency

**Figure 7:**
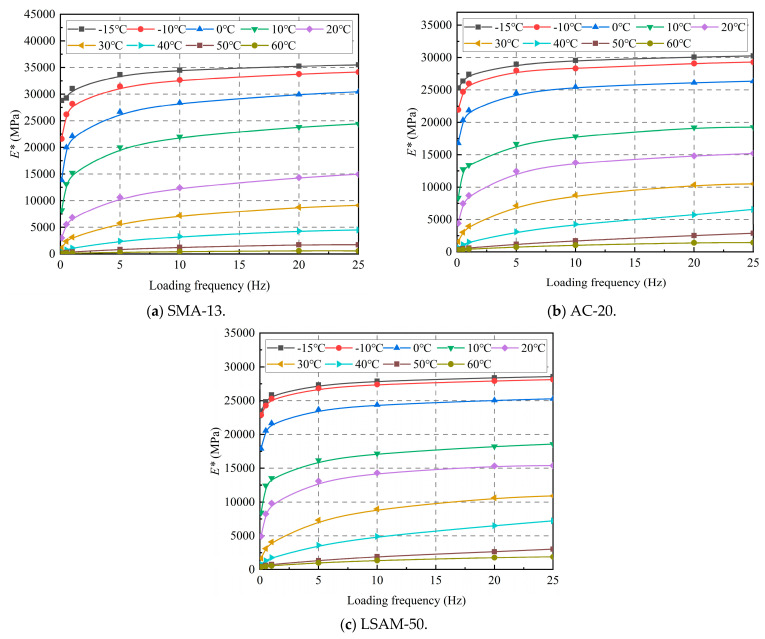
Effect of frequency on dynamic modulus for various LSAM-50 pavement materials.

The effect of loading frequency on the dynamic modulus is shown in Figure 7.

As shown in Figure 7, as the loading frequency increases, the dynamic modulus of the three LSAM-50 pavement materials exhibits a similar growth trend. When the loading frequency is below 5 Hz, the growth rate of the dynamic modulus is more rapid. However, when the loading frequency exceeds 5 Hz, the growth rate of the dynamic modulus significantly slows down, and the dynamic modulus curve gradually begins to plateau.

#### 4.2.3. Effect of Nominal Maximum Particle Size

**Figure 8:**
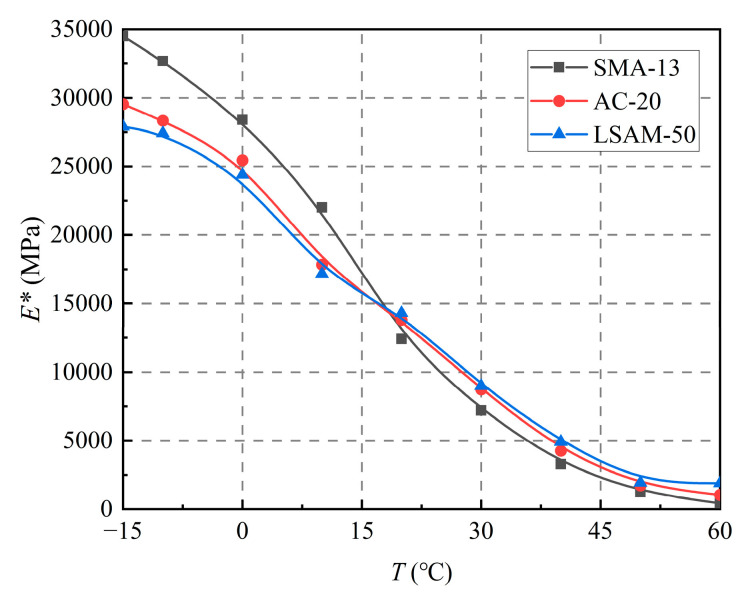
Effect of nominal maximum particle size on dynamic modulus.

The effect of nominal maximum particle size on the dynamic modulus at a loading frequency of 10 Hz is shown in Figure 8.

As shown in Figure 8, the nominal maximum particle size significantly influences the dynamic modulus. When the temperature is below 20 °C, a smaller nominal maximum particle size—indicating a finer gradation—results in a higher dynamic modulus of the asphalt mixture. Conversely, when the temperature exceeds 20 °C, a larger nominal maximum particle size—indicating a coarser gradation—leads to an increased dynamic modulus. To analyze this phenomenon, it is important to note that fine-graded asphalt mixtures typically contain a higher asphalt content. The low-temperature strength of these mixtures primarily depends on the bonding force of the asphalt. In contrast, at elevated temperatures, the bonding force diminishes as the asphalt approaches its softening point. At this stage, the strength of the asphalt mixture is predominantly supported by the interlocking forces of the aggregate skeleton. Therefore, a stronger skeleton correlates with a higher dynamic modulus at elevated temperatures.

### 4.3. Construction of the Dynamic Modulus Master Curve for LSAM-50 Pavement Materials

Due to experimental constraints, directly measuring the dynamic modulus of asphalt mixtures under diverse operational conditions is unfeasible. Nevertheless, the development of a dynamic modulus master curve enables the prediction of mixture behavior over extended temperature and frequency ranges.

In this paper, the dynamic modulus master curves for LSAM-50 pavement materials are developed using the generalized logarithmic sigmoidal model [32], as defined by Equation (9).

(9) $$\mathrm{lg}\left|E^{*}\right|=\delta +\frac{\left(\alpha -\delta \right)}{1+e^{\beta +\gamma \mathrm{lg}fr}}.$$

where α and δ represent the logarithmic maximum and minimum dynamic modulus (MPa); *β* and *γ* represent the shape parameters of the function.

E*max is estimated based on the Hirsh model, as shown in Equations (10) and (11).

(10) $$\alpha =p_{c}\left[4.2\times 10^{6}(1-\frac{VMA}{100})+4.35\times 10^{5}(\frac{VFA\times VMA}{10000}\right]+\frac{1-p_{c}}{\left[\frac{\left(1-\frac{VMA}{100}\right)}{4.2\times 10^{6}}+\frac{VMA}{4.35\times 10^{5}\times VFA}\right]}.$$

(11) $$p_{c}=\frac{{\left[20+\frac{4.35\times 10^{5}VFA}{VMA}\right]}^{0.58}}{{\left[650+\frac{4.35\times 10^{5}VFA}{VMA}\right]}^{0.58}}.$$

where *VFA* represents asphalt saturation (%); *VMA* represents mineral gap ratio (%).

When constructing the master curve of the dynamic modulus, the principle of time–temperature equivalence is applied. The time modulus curve at a specific temperature is shifted by a certain distance to align with the time modulus curve at the reference temperature. This shifted distance is referred to as the displacement factor, while the frequency corresponding to the dynamic modulus after the shift is known as the curtailment frequency. The relationship between the displacement factor and the reduced frequency is shown in Equation (12).

$$\mathrm{lg}f_{r}=\mathrm{lg}f+\mathrm{lg}\alpha_{T}.$$

where *f*_r_ represents the curtailment frequency (Hz), *f* represents the loading frequency during the test (Hz), *α_T_* represents the displacement factor, a function of temperature.

The Arrhenius equation is utilized to calculate the displacement factor, as demonstrated in Equation (13):

$$\mathrm{lg}(\alpha_{T})=\frac{\Delta Ea}{2.303R}(\frac{1}{T+273.15}-\frac{1}{T_{r}+273.15}).$$

where Δ*E_a_* represents the activation energy of the material (kJ mol^−1^), *R* represents the universal gas constant, which is 8.314 kJ mol^−1^, and *Tr* represents the base temperature (°C).

Based on the aforementioned model, the VMA and VFA of the LSAM-50 pavement materials are first determined and substituted into Equations (10) and (11). This allows for the calculation of E*max and its logarithmic value (α) for the LSAM-50 pavement materials, as shown in Table 6.

**Figure 9:**
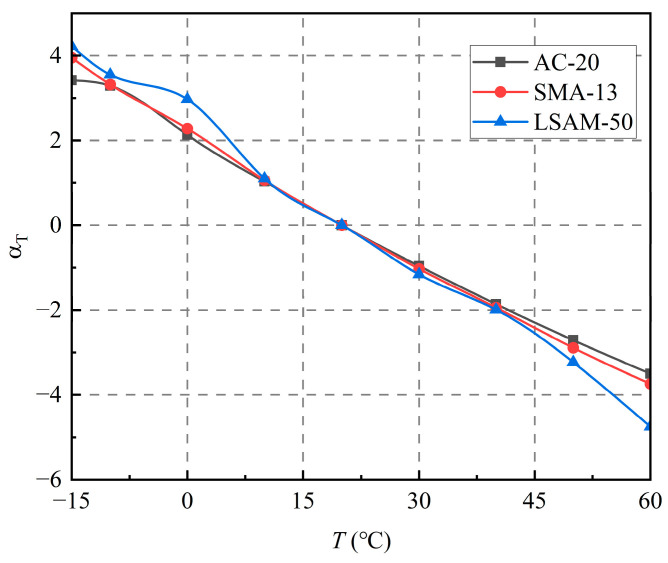
Displacement factor versus temperature curve.

**Figure 10:**
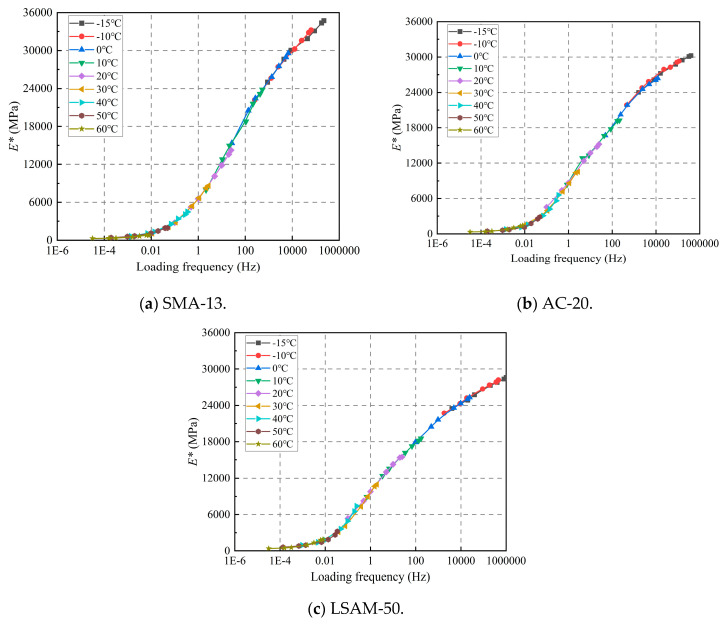
Dynamic modulus master curve for various LSAM-50 pavement materials.

The reference temperature is set to 20 °C (*T_r_*). The logarithmic value (α) of the maximum dynamic modulus is substituted into Equation (9); *T_r_* is substituted into Equation (13). The master curve Equation (10) fitting parameters-δ, β, γ, and Δ*Ea* for the generalized logarithmic sigmoidal model are determined using the least squares method. The calculation results are shown in Table 7. The results of the displacement factor calculations are shown in Table 8. The displacement coefficient versus temperature curves are shown in Figure 9. The master curves and equations for the dynamic modulus are shown in Figure 10 and in Equations (14) through (16).

As shown in Figure 9, the displacement coefficients of the three LSAM-50 pavement materials show a similar trend over the test temperature range (−15 °C to 60 °C), and the ranges of values clearly overlap over the temperature range (10 °C to 45 °C). Overall, the displacement factors of the three LSAM-50 pavement materials differ, but not significantly. The displacement factor is a reflection of the temperature sensitivity of asphalt mixtures, indicating that the overall temperature sensitivity of the three LSAM-50 pavement materials converges. In addition, the similarity of the displacement factors verifies the applicability of the time–temperature equivalence principle (TTSP) in LSAM-50; the temperature–frequency superposition behavior is mainly determined by the asphalt-phase viscoelasticity rather than the aggregate geometric characteristics.

As shown in Figure 10, at a consistent base temperature, the loading frequency similarly affects three LSAM-50 pavement materials, producing a master dynamic modulus curve that resembles an ‘S’ shape. This master curve broadens the range of dynamic modulus and loading frequency, effectively addressing the limitations of laboratory testing.

## 5. Correlation Study Between the Resilient Modulus and Dynamic Modulus

This paper examines the temperature dependence of the modulus of LSAM-50 pavement materials. Additionally, it investigates the correlation between these two moduli, facilitating the prediction of the dynamic modulus based on the resilient modulus. The concept of static rotation guides the physical engineering of road design.

By comparing the resilient modulus of SMA-13, AC-20, and LSAM-50 at various temperatures and frequencies during the dynamic modulus test, it is evident that temperature significantly influences both the resilient modulus and dynamic modulus. This observation suggests a correlation between the two properties. Consequently, this section will treat the resilient modulus, temperature, and loading frequency as independent variables, while the dynamic modulus will be considered the dependent variable. A predictive model for the dynamic modulus of LSAM-50 pavement materials will be established based on these variables (17).

$$E^{*}=A\times E_{c}+B\times T+C\times f+D.$$

where *A*, *B*, *C*, and *D* represent the fitting coefficients.

With the assistance of IBM SPSS Statistics 25 software for multiple regression analysis, the regression coefficients of the dynamic modulus prediction model for LSAM-50 pavement materials are shown in Table 9.

As shown in Table 9, the goodness of fit (*R*^2^) for SMA-13, AC-20, and LSAM-50 are 0.94, 0.95, and 0.95, respectively. The dynamic modulus of the three LSAM-50 pavement materials can be more accurately predicted using Equation (15).

According to the fitting coefficients of Equation (17) and Table 9, the dynamic modulus of the LSAM-50 pavement materials at various temperatures and frequencies has been calculated.

**Figure 11:**
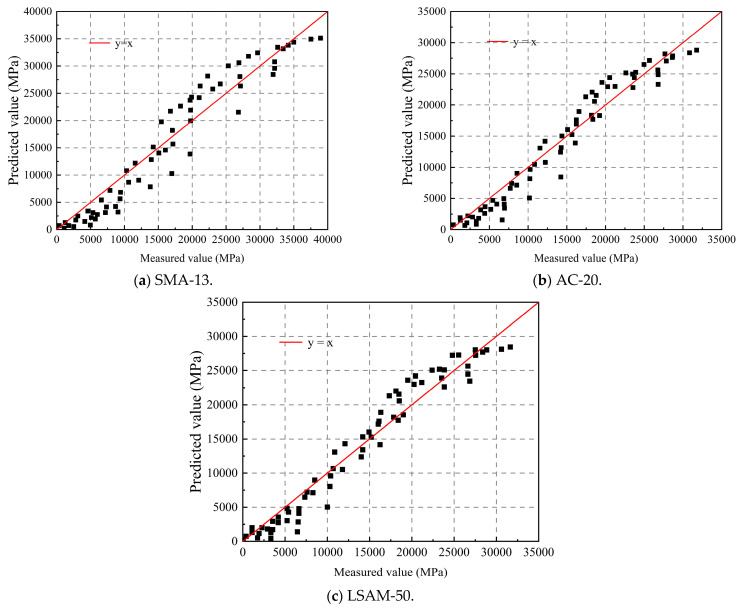
Scatter plot comparing the predicted and measured dynamic modulus for various LSAM-50 pavement materials.

The scatter plot comparing the predicted and measured values of the dynamic modulus for these LSAM-50 pavement materials is presented in Figure 11.

As shown in Figure 10, the dynamic modulus predicted values for the three LSAM-50 pavement materials are plotted against the measured values along the line y = x, which can be utilized to calculate the dynamic modulus as described in Equation (17).

## 6. Conclusions

In this paper, we investigate the modulus temperature dependence of typical structural materials, including SMA-13, AC-20, and LSAM-50, for LSAM-50 pavements. We analyze the factors influencing the modulus, propose a temperature-dependent model for the resilient modulus, and construct the dynamic modulus master curve. Finally, we examine the correlation between the resilient modulus and the dynamic modulus. The main conclusions are as follows:

(1) The resilient modulus of the LSAM-50 pavement materials initially decreases sharply with increasing temperature. When the temperature exceeds 20 °C, the rate of decrease in the resilient modulus diminishes, and the curve of the resilient modulus began to plateau. Additionally, a larger nominal maximum particle size corresponds to a higher resilient modulus of the asphalt mixture. At a temperature of 60 °C, the resilient modulus of LSAM-50 is 1.20 times that of AC-20 and 1.37 times that of SMA-13. Temperature-dependent models for the resilient modulus of the three LSAM-50 pavement materials are established, with correlation coefficients (*R*^2^) exceeding 0.97.
(2) The dynamic modulus of three LSAM-50 pavement materials decreases significantly with increasing temperature, and the rate of decrease diminishes when the temperature exceeds 40 °C. Additionally, the dynamic modulus of three LSAM-50 pavement materials increases with rising loading frequency, and the curve of dynamic modulus changes begins to plateau when the loading frequency exceeds 5 Hz.
(3) The displacement factors of three LSAM-50 pavement materials have been determined. The temperature sensitivities of the three LSAM-50 pavement materials are relatively similar. Additionally, dynamic modulus master curves are constructed for three LSAM-50 pavement materials, extending the dynamic modulus and loading frequency across a broader range and thereby overcoming the limitations of laboratory testing.
(4) Multiple linear regression models for the dynamic modulus of three LSAM-50 pavement materials, based on resilient modulus, temperature, and loading frequency, are developed. The goodness of fit (*R*^2^) values of SMA-13, AC-20, and LSAM-50 are 0.94, 0.95, and 0.95. The dynamic modulus can be predicted using the resilient modulus, offering critical insights for optimizing pavement design in civil engineering.

These findings reveal the effects of temperature on LSAM-50 pavement materials and provide a theoretical foundation for the design of LSAM-50 pavements. However, it is important to emphasize that these results are based on laboratory tests. Further validation through actual road construction projects is necessary to confirm the reliability of the modulus-temperature dependence model for LSAM-50 pavement materials.

## Figures and Tables

**Table 1:** Technical properties of asphalt.

Items		Measured Value	
		70# Base Asphalt	SBS-Modified Asphalt
Density (g/cm^3^)		1.039	1.013
Penetration (0.1 mm)		63	53
Ductility (cm)		38	36
Softening point (°C)		46.8	78.5
After TFTO	Mass change (%)	0.13	−0.327
	Ductility (cm)	9	22
	Penetration ratio (%)	65	73

**Table 2:** Mineral grading of asphalt mixtures.

Sieve Size (mm)		53	37.5	19	9.5	4.75	2.36	1.18	0.6	0.3	0.15	0.075
Passing ratio (by mass) (%)	SMA-13	100	100	100	58.8	25.0	20.5	17.0	14.5	13.0	12.0	9.0
	AC-20	100	100	95.0	50.0	33.0	23.0	16.0	12.0	9.0	7.0	5.0
	LSAM-50	100	70.0	60.0	42.0	34.0	26.0	18.0	14.0	10.0	7.5	4.5

**Table 3:** *E_c_*_0_ of LSAM-50 pavement materials.

Type of Asphalt Mixture	SMA-13	AC-20	LSAM-50
*E*_c0_ (MPa)	3066	3511	4034

**Table 4:** Ec~*T* fitted regression coefficients b.

Type of Asphalt Mixture	SMA-13	AC-20	LSAM-50
*b*	−0.039	−0.039	−0.039
*R* ^2^	0.97	0.98	0.98

**Table 5:** Measured values of dynamic modulus.

Type of Asphalt Mixture	*T* (°C)	The Following Loading Frequencies (Hz) Correspond to the Dynamic Modulus (MPa)						
		25	20	10	5	1	0.5	0.1
SMA-13	−15	35,515	35,255	34,507	33,678	31,071	29,270	28,817
	−10	34,164	33,800	32,677	31,463	28,210	26,215	21,626
	0	30,465	29,940	28,400	26,685	22,133	20,008	13,856
	10	24,429	23,842	22,010	20,044	15,249	13,118	8206
	20	14,966	14,337	12,414	10,649	6853	5566	3013
	30	9132	8765	7206	5807	3149	2370	1161
	40	4504	4308	3278	2412	1108	783	390.5
	50	1731	1728	1239	855.2	378.5	274.2	164.9
	60	567	598	427	290	146	122	96
AC-20	−15	30,246	30,057	29,542	28,957	27,424	26,373	25,280
	−10	29,296	29,097	28,329	28,006	25,987	24,714	21,933
	0	26,344	26,116	25,451	24,529	21,844	20,318	16,782
	10	19,253	19,213	17,833	16,673	13,389	12,757	8337
	20	15,201	14,824	13,790	12,449	8685	7455	4420
	30	10,511	10,352	8738	7129	3972	3024	1604
	40	6566	5718	4263	3088	1517	1113	653
	50	2874	2523	1719	1169	604	509	372
	60	1448	1415	1039	756	415	353	285
LSAM-50	−15	28,573	28,392	27,899	27,341	25,854	24,833	23,454
	−10	28,120	27,924	27,407	26,839	25,313	24,281	22,883
	0	25,277	25,050	24,384	23,683	21,652	20,547	17,881
	10	18,561	18,224	17,173	16,168	13,535	12,420	8440
	20	15,388	15,330	14,294	13,086	9823	8243	4936
	30	10,920	10,634	8982	7330	4087	3113	1679
	40	7217	6513	4915	3605	1763	1302	786
	50	3028	2642	1936	1313	747	611	442
	60	1882	1772	1333	988	559	477	374

**Table 6:** Calculation results of E*max.

Type of Asphalt Mixture	*VMA* (%)	*VFA* (%)	E*max (MPa)	α (MPa)
SMA-13	17.2	77.5	3,265,791	6.51
AC-20	13.0	70.1	3,446,116	6.53
LSAM-50	7.8	51.3	3,666,467	6.56

**Table 7:** Regression coefficients of the dynamic modulus master curve.

Type of Asphalt Mixture	Regression Coefficient			
	*δ*	*β*	*γ*	Δ*Ea*
SMA-13	3.07	0.15	0.96	194,989
AC-20	3.04	0.03	0.90	202,109
LSAM-50	4.72	−1.59	−0.49	326,538

**Table 8:** Dynamic modulus master curve displacement factor (α_T_).

Type of Asphalt Mixture	Temperature (°C)								
	−15	−10	0	10	20	30	40	50	60
SMA-13	3.42	3.29	2.13	1.03	0	−0.96	−1.86	−2.71	−3.50
AC-20	3.95	3.32	2.28	1.06	0	−1.03	−1.94	−2.89	−3.74
LSAM-50	4.22	3.55	2.97	1.10	0	−1.16	−1.99	−3.23	−4.75

**Table 9:** Regression coefficients of the dynamic modulus prediction model.

Type of Asphalt Mixture	*A*	*B*	*C*	*D*	*R* ^2^
SMA-13	3.422	−248	289	9145	0.94
AC-20	1.976	−266	229	11,879	0.95
LSAM-50	1.206	−281	205	13,576	0.95

## Data Availability

The original contributions presented in this study are included in the article. Further inquiries can be directed to the corresponding author.

## Nomenclature

LSAM-50 pavement	LSAM-50 flexible base asphalt pavement
SBS	Styrene–butadiene–styrene
*E*_c_	Resilient modulus
*E**	Dynamic modulus
*T*	Temperature
*f*	Loading frequencies
*f_r_*	Curtailment frequency
α	Logarithmic maximum dynamic modulus
δ	Logarithmic minmum dynamic modulus
$\alpha_{T}$	Displacement factor
